# Photocatalytic reduction synthesis of SrTiO_3_-graphene nanocomposites and their enhanced photocatalytic activity

**DOI:** 10.1186/1556-276X-9-327

**Published:** 2014-06-29

**Authors:** Tao Xian, Hua Yang, Lijing Di, Jinyuan Ma, Haimin Zhang, Jianfeng Dai

**Affiliations:** 1 State Key Laboratory of Advanced Processing and Recycling of Non-ferrous Metals, Lanzhou University of Technology, Lanzhou 730050, People’s Republic of China; 2 School of Science, Lanzhou University of Technology, Lanzhou 730050, People’s Republic of China

**Keywords:** SrTiO_3_-graphene nanocomposites, Photocatalysis, Photocatalytic mechanism

## Abstract

SrTiO_3_-graphene nanocomposites were prepared via photocatalytic reduction of graphene oxide by UV light-irradiated SrTiO_3_ nanoparticles. Fourier transformed infrared spectroscopy analysis indicates that graphene oxide is reduced into graphene. Transmission electron microscope observation shows that SrTiO_3_ nanoparticles are well assembled onto graphene sheets. The photocatalytic activity of as-prepared SrTiO_3_-graphene composites was evaluated by the degradation of acid orange 7 (AO7) under a 254-nm UV irradiation, revealing that the composites exhibit significantly enhanced photocatalytic activity compared to the bare SrTiO_3_ nanoparticles. This can be explained by the fact that photogenerated electrons are captured by graphene, leading to an increased separation and availability of electrons and holes for the photocatalytic reaction. Hydroxyl (·OH) radicals were detected by the photoluminescence technique using terephthalic acid as a probe molecule and were found to be produced over the irradiated SrTiO_3_ nanoparticles and SrTiO_3_-graphene composites; especially, an enhanced yield is observed for the latter. The influence of ethanol, KI, and N_2_ on the photocatalytic efficiency was also investigated. Based on the experimental results, ·OH, h^+^, and H_2_O_2_ are suggested to be the main active species in the photocatalytic degradation of AO7 by SrTiO_3_-graphene composites.

**PACS:**

61.46. + w; 78.67.Bf; 78.66.Sq

## Background

Semiconductor photocatalysts have attracted considerable attention over the past decades due to their potential applications in solar energy conversion and environmental purification [1,2]. Among them, SrTiO_3_, a well-known cubic perovskite-type multimetallic oxide with a bandgap energy (*E*_g_) of approximately 3.2 eV, is proved to be a promising photocatalyst for water splitting and degradation of organic pollutants [3-6]. Furthermore, the photocatalytic activity of SrTiO_3_ can be tailored or enhanced by doping with metalloid elements, decoration with noble metals, and composite with other semiconductors [7-10]. It is generally accepted that the basic principle of semiconductor photocatalysis involves the photogeneration of electron–hole (e^-^-h^+^) pairs, migration of the photogenerated carriers to the photocatalyst surface, redox reaction of the carriers with other chemical species to produce active species (such as · OH, ·O_2_, and H_2_O_2_), and attack of the active species on pollutants leading to their degradation. In these processes, the high recombination rate of the photogenerated carries greatly limits the photocatalytic activity of catalysts. Therefore, the effective separation of photogenerated electron–hole pairs is very important in improving the photocatalytic efficiency.

Graphene, being a two-dimensional (2D) sheet of *sp*^2^-hybridized carbon atoms, possesses unique properties including high electrical conductivity, electron mobility, thermal conductivity, mechanical strength, and chemical stability [11-13]. On account of its outstanding properties, graphene has been frequently used as an ideal support to integrate with a large number of functional nanomaterials to form nanocomposites with improved performances in the fields of photocatalysts [14-21], supercapacitors [22], field-emission emitters [23], and fuel cells [24]. Particularly, the combination of graphene with photocatalysts is demonstrated to be an efficient way to promote the separation of photogenerated electron–hole pairs and then enhance their photocatalytic activity [14-21]. In these photocatalyst-graphene composites, photogenerated electrons can be readily captured by graphene which acts as an electron acceptor, leading to an increasing availability of photogenerated electrons and holes participating in the photocatalytic reactions. But so far, the investigation concerning the photocatalytic performance of SrTiO_3_-graphene nanocomposites has been rarely reported.

Up to now, semiconductor-graphene nanocomposites have been generally prepared using graphene oxide as the precursor, followed by its reduction to graphene. To reduce the graphene oxide, several methods have been employed including chemical reduction using hydrazine or NaBH_4_[14], high-temperature annealing reduction [15], hydrothermal reduction using supercritical water [16], green chemistry method [17], and photocatalytic reduction using semiconductors [18-21]. Among them, the photocatalytic reduction is an environment-friendly and a mild way for the synthesis of semiconductor-graphene composites. In this route, the solution containing the photocatalyst and graphene oxide is irradiated with light energy greater than the *E*_g_ of the photocatalyst, during which graphene oxide receives electrons from the excited photocatalyst and is thus reduced to graphene. During the photocatalytic reduction process, photocatalyst nanoparticles are assembled onto graphene sheets to form photocatalyst-graphene composites. Herein, we report the synthesis of SrTiO_3_-graphene nanocomposites via the photocatalytic reduction method. The photocatalytic activity of the composites was evaluated by the degradation of acid orange 7 (AO7) under ultraviolet (UV) light irradiation, and the photocatalytic mechanism involved was discussed.

## Methods

SrTiO_3_ nanoparticles were synthesized via a polyacrylamide gel route as described in the literature [25]. The graphene oxide used in this research was purchased from Nan-Jing XF Nano Materials Tech Co. Ltd. (Nanjing, China). SrTiO_3_-graphene composites were prepared via a photocatalytic reduction route. A certain amount of graphene oxide was dispersed in 50 mL distilled water, followed by ultrasonic treatment of the suspension for 30 min. Then, 0.1 g SrTiO_3_ nanoparticles and 0.0125 g ammonium oxalate (AO) were added to the suspension under magnetic stirring. After stirring for 10 min, the mixture was purged with nitrogen and exposed to UV light irradiation from a 15-W low-pressure mercury lamp for 5 h under mild stirring. During the irradiation, the color of the mixture changed from brown to black, indicating the reduction of the graphene oxide. After that, the product was separated from the reaction solution by centrifugation at 4,000 rpm for 10 min, washed several times with distilled water and absolute ethanol, and then dried in a thermostat drying oven at 60°C for 4 h to obtain SrTiO_3_-graphene composites. A series of samples were prepared by varying the weight fraction of graphene oxide from 2.5% to 10%.

The photocatalytic activity of the samples was evaluated by the degradation of AO7 under UV light irradiation of a 15-W low-pressure mercury lamp (*λ* = 254 nm). The initial AO7 concentration was 5 mg L^-1^ with a photocatalyst loading of 0.5 g L^-1^. Prior to irradiation, the mixed solution was ultrasonically treated in the dark to make the photocatalyst uniformly dispersed. The concentration of AO7 after the photocatalytic degradation was determined by measuring the absorbance of the solution at a fixed wavelength of 484 nm. Before the absorbance measurements, the reaction solution was centrifuged for 10 min at 4,000 rpm to remove the photocatalyst. The degradation percentage is defined as (*C*_0_ - *C*_t_) / *C*_0_ × 100%, where *C*_0_ and *C*_t_ are the AO7 concentrations before and after irradiation, respectively. To investigate the photocatalytic stability of the SrTiO_3_-graphene composites, the recycling tests for the degradation of AO7 using the composite were carried out. After the first cycle, the photocatalyst was collected by centrifugation, washed with water, and dried. The recovered photocatalyst was introduced to the fresh AO7 solution for the next cycle of the photocatalysis experiment under the same conditions. The process was repeated four times.

Terephthalic acid (TA) was used as a probe molecule to examine hydroxyl (·OH) radicals produced over the irradiated SrTiO_3_-graphene composites. It is expected that TA reacts with · OH to generate a highly fluorescent compound, 2-hydroxyterephthalic acid (TAOH). By measuring the photoluminescence (PL) intensity of TAOH that is pronounced around 429 nm, the information about · OH can be obtained. TA was dissolved in a NaOH solution (1.0 mmol L^-1^) to make a 0.25-mmol L^-1^ TA solution and then to the solution was added 0.5 g L^-1^ SrTiO_3_-graphene composites. The mixed solution, after several minutes of ultrasound treatment in the dark, was illuminated under a 15-W low-pressure mercury lamp. The reacted solution was centrifuged for 10 min at 4,000 rpm to remove the photocatalyst and was then used for the PL measurements through a fluorescence spectrophotometer with the excitation wavelength of 315 nm.

The phase purity of the samples was examined by means of X-ray powder diffraction (XRD) with Cu Kα radiation. Fourier transform infrared spectroscopy (FTIR) measurements were carried out on a Bruker IFS 66v/S spectrometer (Ettlingen, Germany). The morphology of the samples was observed by a field emission transmission electron microscope (TEM). The UV-visible diffuse reflectance spectra were measured using a UV-visible spectrophotometer with an integrating sphere attachment.

## Results and discussion

**Figure 1:**
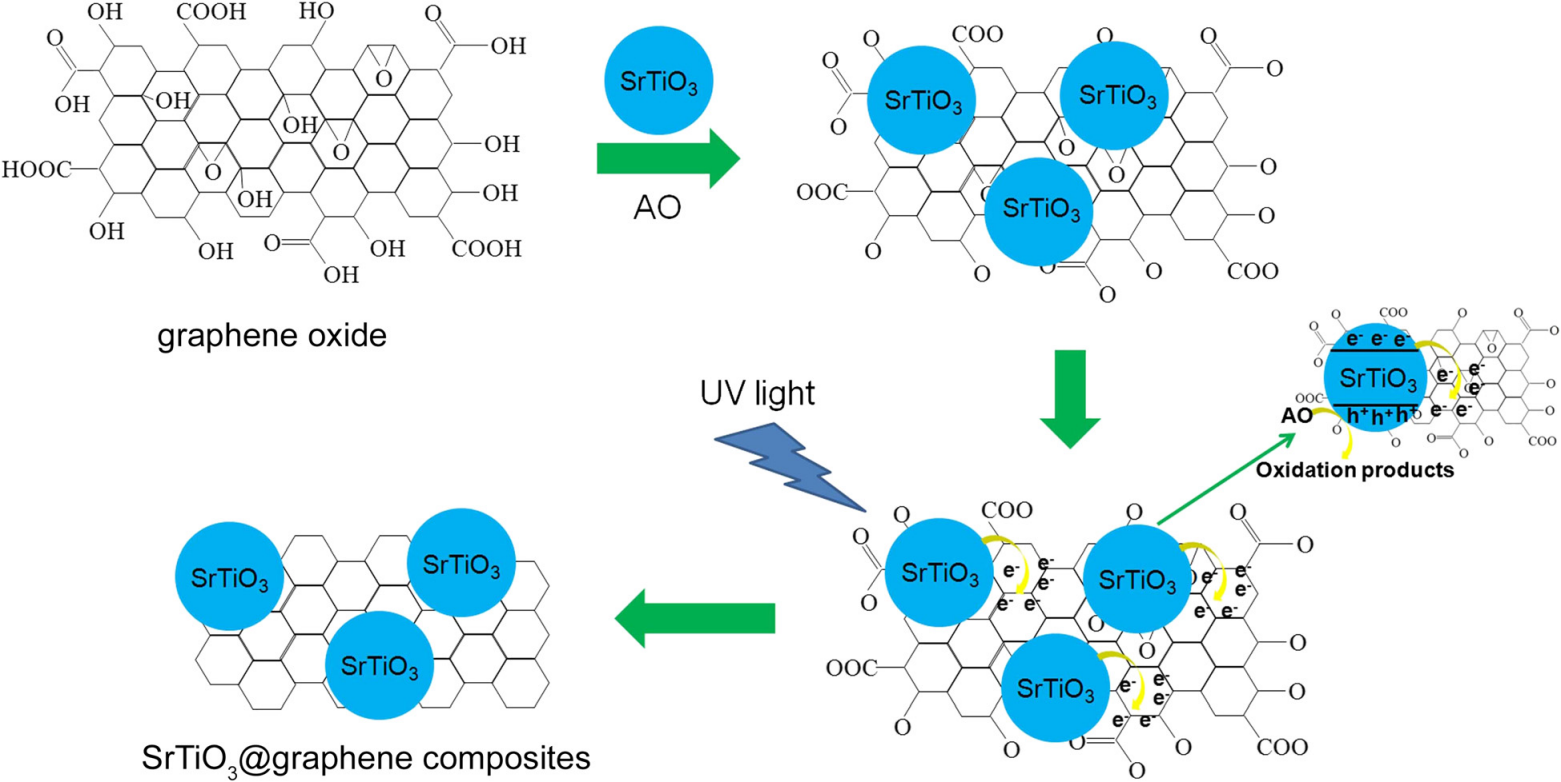
**Schematic illustration of the photocatalytic reduction process of graphene oxide by UV light-irradiated SrTiO**_**3 **_**nanoparticles.**

Figure 1 schematically shows the photocatalytic reduction process of graphene oxide by UV light-irradiated SrTiO_3_ nanoparticles. It is noted that the SrTiO_3_ particles have an isoelectric point at pH 8.5 [26]; that is, they bear a negative surface charge when pH > 8.5 and a positive surface charge when pH < 8.5. When the SrTiO_3_ particles are added to the graphene oxide suspension, the pH value of the mixture is measured to be approximately 6.5, implying that the SrTiO_3_ particle surface is positively charged. On the other hand, the oxygen-containing functional groups of graphene oxide (such as carboxylic acid -COOH and hydroxyl -OH) are deprotonated when it immersed in water, which leads to negative charges created on graphene oxide [27]. As a result, the SrTiO_3_ particles are expected to be adsorbed onto the graphene oxide sheets through electrostatic interactions. Upon UV-light irradiation, electrons and holes are produced on the conduction band (CB) and valence band (VB) of the SrTiO_3_ particles, respectively. The photogenerated holes are captured by ammonium oxalate that is a hole scavenger [28], leaving behind the photogenerated electrons on the surface of the SrTiO_3_ particles. The electrons are injected from the SrTiO_3_ particles into the graphene oxide and react with its oxygen-containing functional groups to reduce graphene oxide.

**Figure 2:**
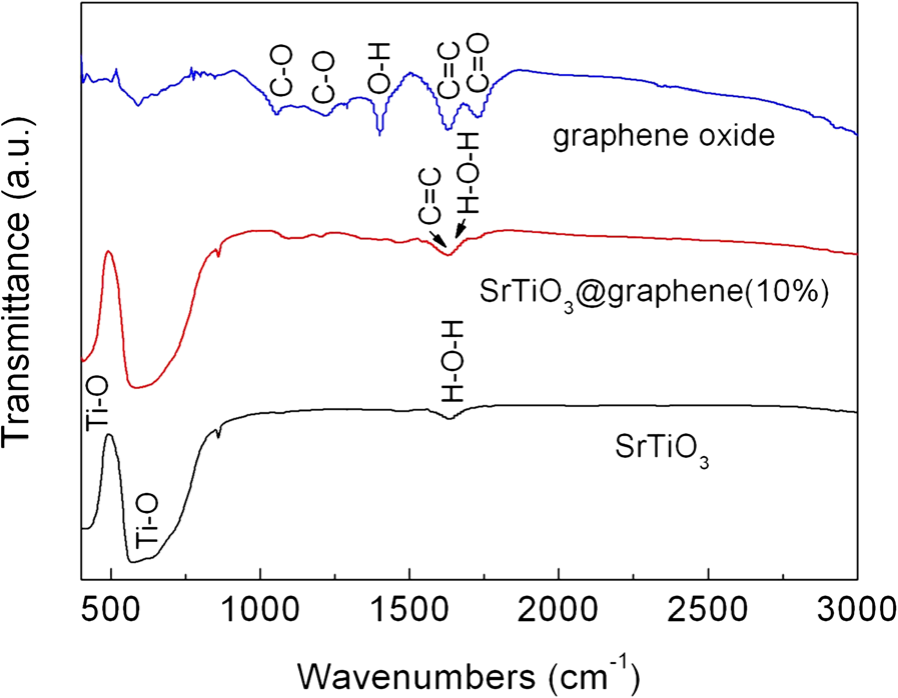
**FTIR spectra of graphene oxide, SrTiO**_**3 **_**particles, and SrTiO**_**3**_**-graphene(10%) composites.**

Figure 2 shows the FTIR spectra of graphene oxide, SrTiO_3_ particles, and SrTiO_3_-graphene(10%) composites. In the spectrum of graphene oxide, the absorption peak at 1,726 cm^-1^ is caused by the C = O stretching vibration of the COOH group. The peak at 1,620 cm^-1^ is attributed to the C = C skeletal vibration of the graphene sheets. The absorption peak of O-H deformation vibrations in C-OH can be seen at 1,396 cm^-1^. The absorption bands at around 1,224 and 1,050 cm^-1^ are assigned to the C-O stretching vibration. For the SrTiO_3_ particles, the broad absorption bands at around 447 and 625 cm^-1^ correspond to TiO_6_ octahedron bending and stretching vibration, respectively [29]. The absorption peak at around 1,630 cm^-1^ is due to the bending vibration of H-O-H from the adsorbed H_2_O. In the spectrum of the SrTiO_3_-graphene composites, the characteristic peaks of SrTiO_3_ are detected. The absorption peak at 1,630 cm^-1^ is the overlay of the vibration peak of H-O-H from H_2_O and C = C skeletal vibration peak in the graphene sheets. However, the absorption peaks of oxygen-containing functional groups, being characteristic for graphene oxide, disappear. The results demonstrate that graphene oxide is completely reduced to graphene during the photocatalytic reduction process.

**Figure 3:**
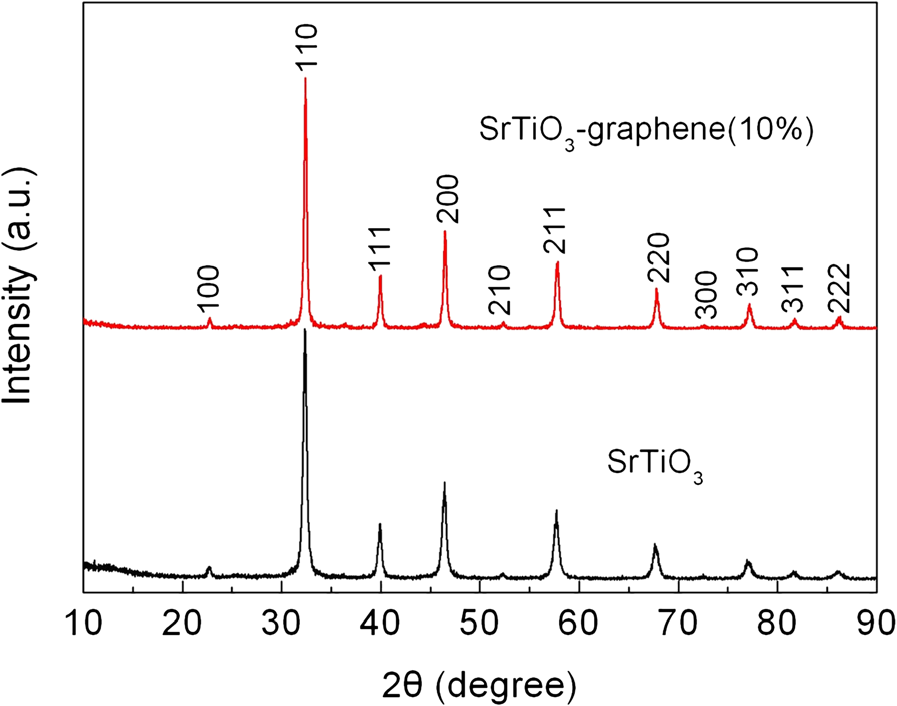
**XRD patterns of the SrTiO**_**3 **_**particles and SrTiO**_**3**_**-graphene(10%) composites.**

Figure 3 shows the XRD patterns of the SrTiO_3_ particles and the SrTiO_3_-graphene (10%) composites. It is seen that all the diffraction peaks for the bare SrTiO_3_ particles and the composites can be index to the cubic structure of SrTiO_3_, and no traces of impurity phases are detected. This indicates that the SrTiO_3_ particles undergo no structural change after the photocatalytic reduction of graphene oxide. In addition, no apparent diffraction peaks of graphene in the composites are observed, which is due to the low content and relatively weak diffraction intensity of the graphene.

**Figure 4:**
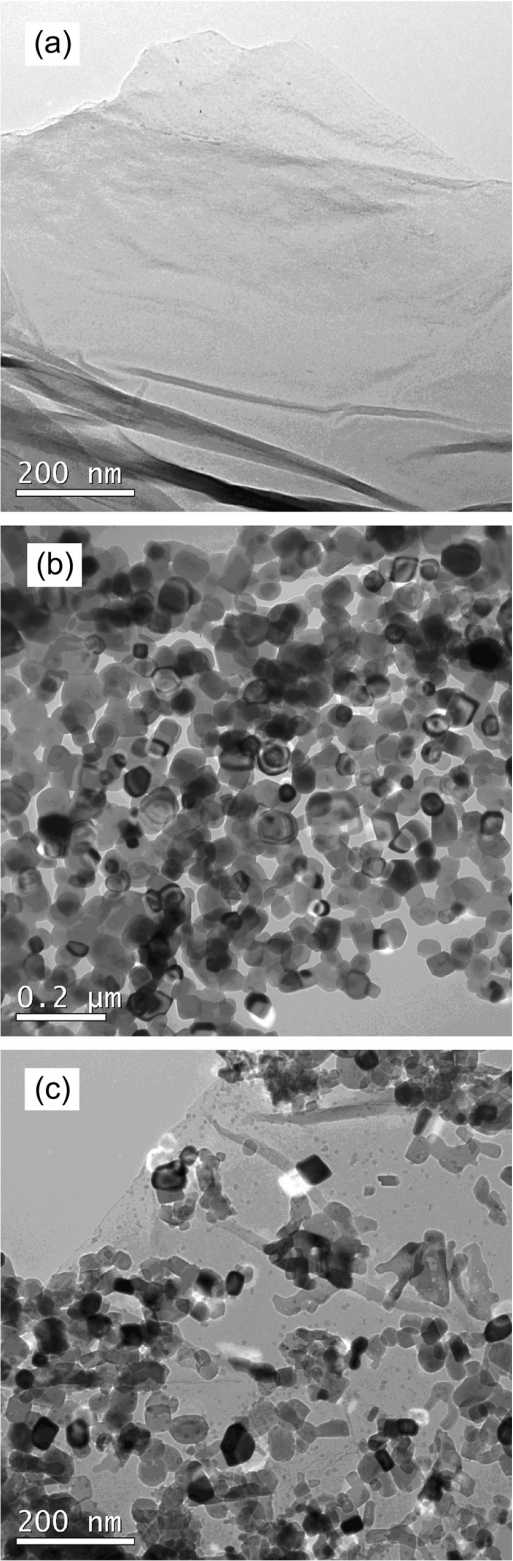
**TEM images of (a) graphene oxide, (b) SrTiO**_**3 **_**particles, and (c) SrTiO**_**3**_**-graphene(10%) composites.**

Figure 4a shows the TEM image of graphene oxide, indicating that it has a typical two-dimensional sheet structure with crumpled feature. Figure 4b shows the TEM image of the SrTiO_3_ particles, revealing that the particles are nearly spherical in shape with an average size of about 55 nm. The TEM image of the SrTiO_3_-graphene(10%) composites is presented in Figure 4c, from which one can see that the SrTiO_3_ particles are well assembled onto the graphene sheet.

**Figure 5:**
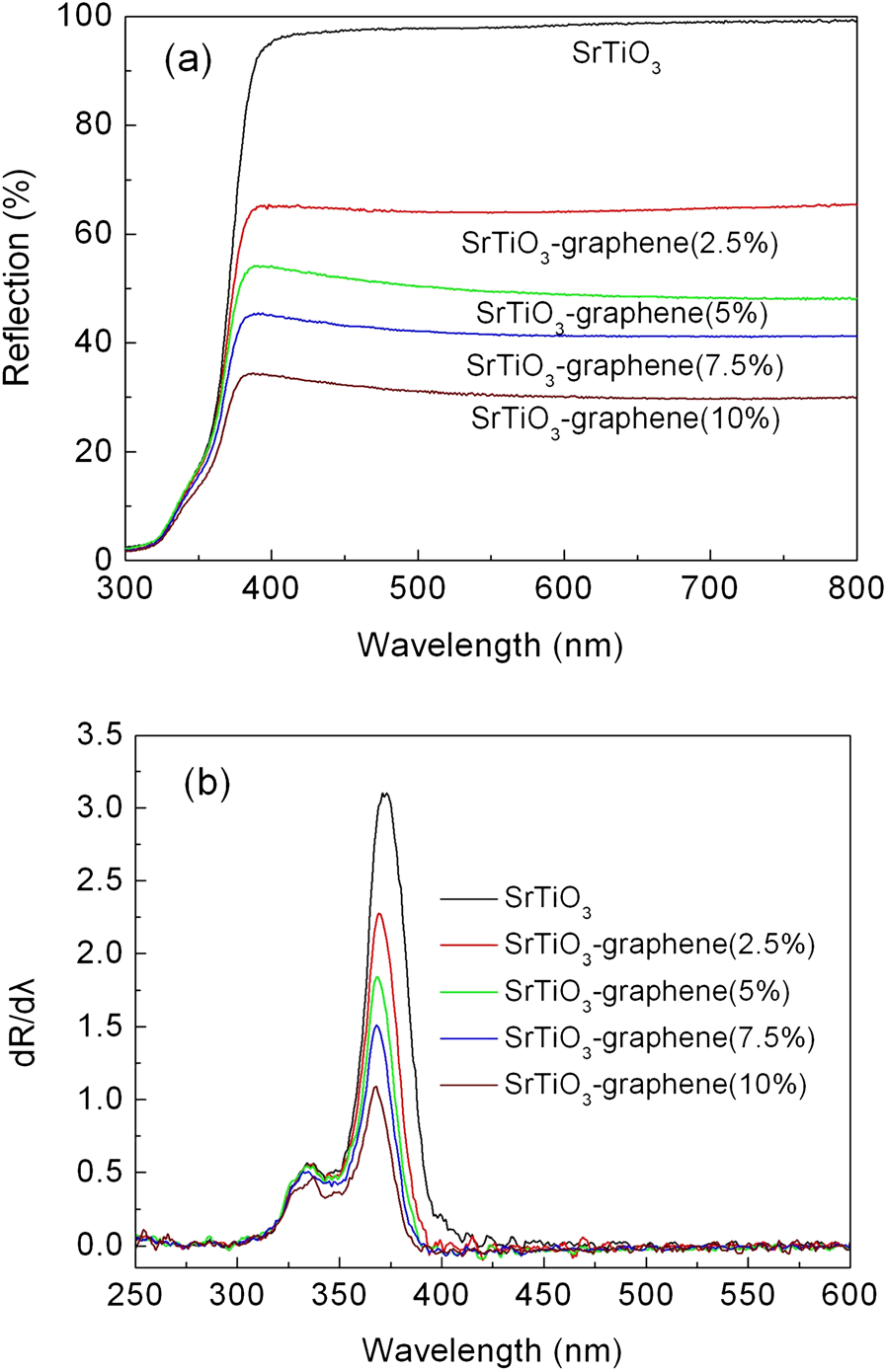
**Diffuse reflectance spectra and corresponding first derivative. (a)** Diffuse reflectance spectra of the samples. **(b)** Corresponding first derivative of diffuse reflectance spectra.

Figure 5a shows the UV-visible diffuse reflectance spectra of the SrTiO_3_ particles and SrTiO_3_-graphene composites. The composites display continuously enhanced light absorbance over the whole wavelength range with increasing graphene content. This can be attributed to the strong light absorption of graphene in the UV-visible light region [30]. Figure 5b shows the corresponding first derivative of the reflectance (*R*) with respect to wavelength *λ* (i.e., *dR* / *dλ*), where the peak wavelength is characterized to be the absorption edge of the samples. It is seen that the SrTiO_3_ particles and composites present two absorption peaks in the derivative spectra. The strong and sharp absorption edge at approximately 370 nm is suggested to be attributed to the electron transition from valence band to conduction band. In comparison to the SrTiO_3_ particles, the SrTiO_3_-graphene composites show almost no shift in this absorption edge, indicating that the effect of graphene on the band structure of SrTiO_3_ can be neglected. From this absorption edge, the *E*_g_ of the samples is obtained to be approximately 3.35 eV. In addition, the relatively weak absorption edge at approximately 335 nm may be ascribed to the surface effects.

**Figure 6:**
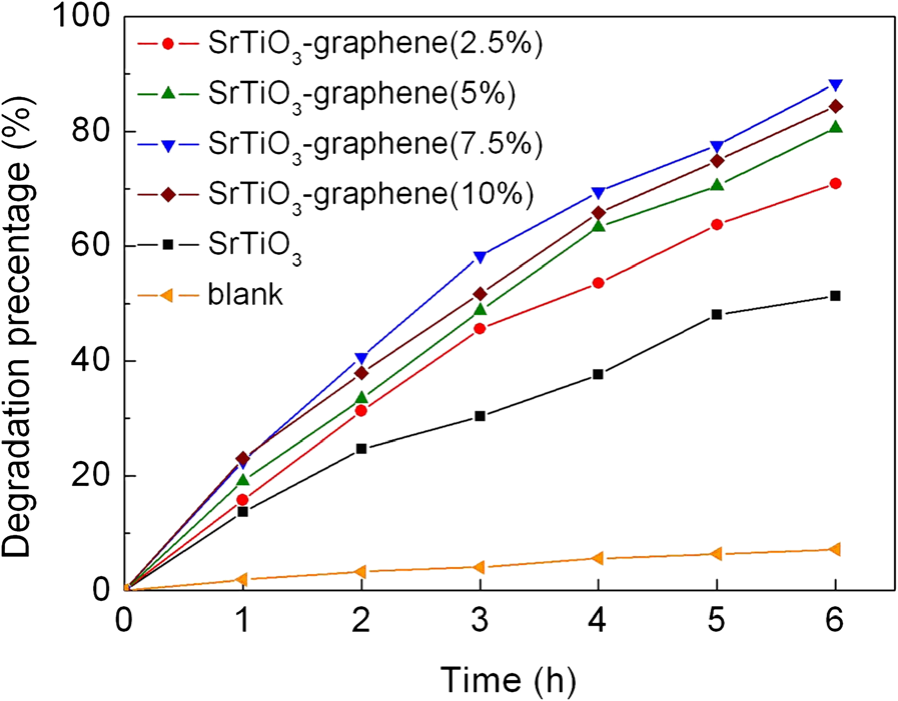
**Photocatalytic degradation of AO7 over SrTiO**_**3 **_**particles and SrTiO**_**3**_**-graphene composites.** This degradation is a function of irradiation time, along with the blank experiment result.

The photocatalytic activity of the SrTiO_3_-graphene composites was evaluated by the degradation of AO7 under UV light irradiation. Figure 6 shows the photocatalytic degradation of AO7 over the SrTiO_3_-graphene composites as a function of irradiation time (*t*). The blank experiment result is also shown in Figure 6, from which one can see that AO7 is hardly degraded under UV light irradiation without photocatalysts, and its degradation percentage is less than 8% after 6 h of exposure. After the 6-h irradiation in the presence of SrTiO_3_ particles, about 51% of AO7 is observed to be degraded. When the SrTiO_3_ particles assembled on the graphene sheets, the obtained samples exhibit higher photocatalytic activity than the bare SrTiO_3_ particles. In these composites, the photocatalytic activity increases gradually with increasing graphene content and achieves the highest value when the content of graphene reaches 7.5%, where the degradation of AO7 is about 88% after irradiation for 6 h. Further increase in graphene content leads to the decrease of the photocatalytic activity.

**Figure 7:**
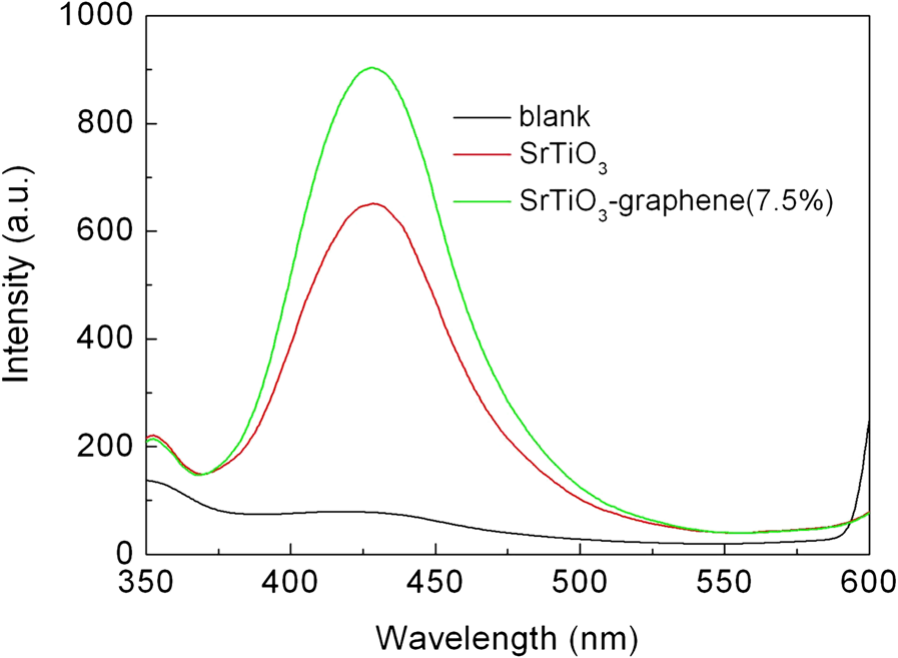
**PL spectra of the TA solution after reacting for 6 h over the irradiated samples.** The blank experiment result is also shown.

Figure 7 shows the PL spectra of the TA solution after reacting for 6 h over the UV light-irradiated SrTiO_3_ particles and SrTiO_3_-graphene(7.5%) composites. The blank experiment result indicates almost no PL signal at 429 nm after irradiation without photocatalyst. On irradiation in the presence of the SrTiO_3_ particles, the PL signal centered around 429 nm is obviously detected, revealing the generation of · OH radicals. When the SrTiO_3_-graphene composites are used as the photocatalyst, the PL signal becomes more intense, suggesting that the yield of the · OH radicals is enhanced over the irradiated composites.

**Figure 8:**
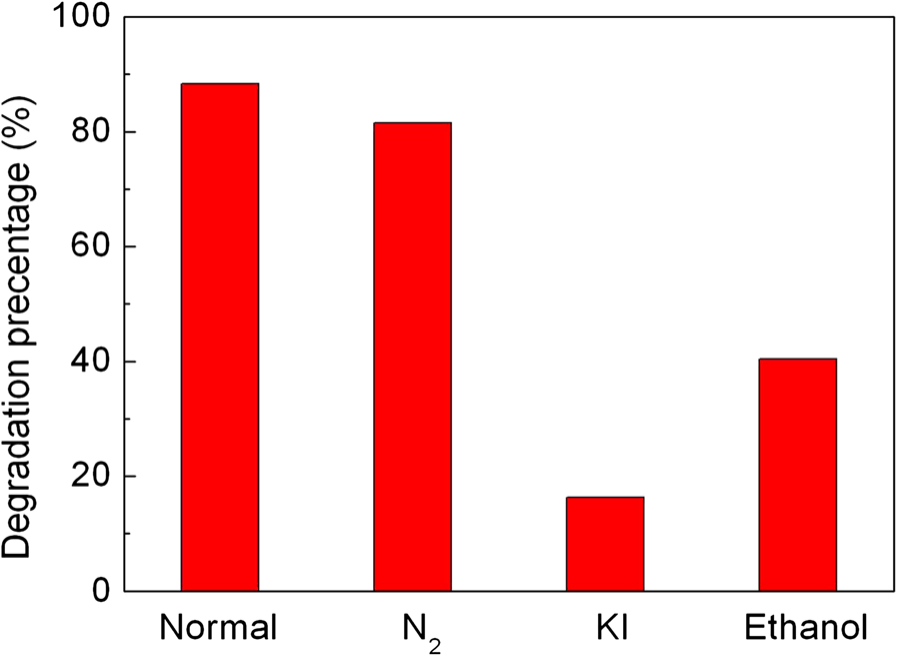
**Effects of N**_**2**_**, ethanol, and KI on the degradation percentage of AO7 over SrTiO**_**3**_**-graphene(7.5%) composites.** The irradiation time is 6 h.

Generally, h^+^, ·OH, ·O_2_, and H_2_O_2_ are thought to be the main active species responsible for the dye degradation [31]. It is known that ethanol is a scavenger for · OH, and KI is a scavenger for both · OH and h^+^[32,33]. By investigating the effect of ethanol and KI on the photocatalytic efficiency of the composites toward the AO7 degradation, we can clarify the role of h^+^ and · OH in the photocatalysis. The role of · O_2_ and H_2_O_2_, which are derived from the reaction between dissolved O_2_ and photogenerated e^-^, on the dye degradation can be examined by investigating the effect of N_2_ on the photocatalytic efficiency since the dissolved O_2_ can be removed from the solution by the N_2_-purging procedure. Figure 8 shows the effect of N_2_ (bubbled at a rate of 0.1 L min^-1^), ethanol (10% by volume), and KI (2 × 10^-3^ mol L^-1^) on the degradation percentage of AO7 after 6 h of photocatalysis. It is demonstrated that when adding ethanol to the reaction solution, the photocatalytic degradation of AO7 undergoes a substantial decrease, from approximately 88% under normal condition to approximately 40% on addition of ethanol. This suggests that · OH radical is an important active species responsible for the dye degradation. Figure 7 provides direct evidence showing the generation of · OH radicals over the irradiated SrTiO_3_-graphene composites. The addition of KI to the reaction solution results in a higher suppression of the photocatalytic efficiency compared to the addition of ethanol, where only 16% of AO7 is caused to be degraded, indicating that the photogenerated h^+^ also plays a role in the degradation of AO7. In addition, the photocatalytic efficiency decreases slightly under N_2_-purging condition, implying comparatively minor role of · O_2_ and/or H_2_O_2_ for the dye degradation.

In order to understand the photocatalytic mechanism of semiconductor-based photocatalysts, it is essential to determine their energy-band potentials since the redox ability of photogenerated carriers is associated with energy-band potentials of photocatalysts. The conduction band and valence band potentials of SrTiO_3_ can be calculated using the following relation [34]:

(1) $$\begin{array}{l} E_{\text{CB}}=X-E^{e}-\;0.5E_{g}\\ E_{\text{VB}}=E_{\mathrm{CB}}+E_{g}, \end{array}$$

where *X* is the absolute electronegativity of SrTiO_3_ (defined as the arithmetic mean of the electron affinity and the first ionization of the constituent atoms) and estimated to be 5.34 eV according to the data reported in the literature [35,36], *E*^e^ is the energy of free electrons on the hydrogen scale (4.5 eV), and *E*_g_ is the bandgap energy of SrTiO_3_ (3.35 eV). The conduction band and valence band potentials of SrTiO_3_ vs. normal hydrogen electrode (NHE) are therefore calculated to be *E*_CB_ = -0.84 V and *E*_VB_ = +2.51 V, respectively.

**Figure 9:**
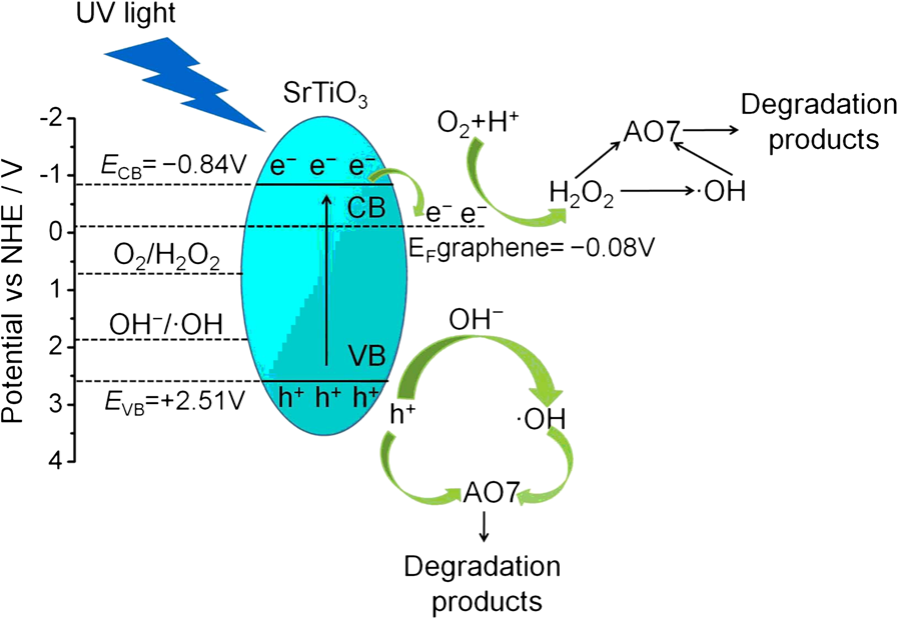
**Schematic illustration of the photocatalytic mechanism of SrTiO**_**3**_**-graphene composites toward the degradation of AO7.**

Based on the obtained experimental results, a possible photocatalytic mechanism of SrTiO_3_-graphene composites toward the degradation of AO7 is schematically shown in Figure 9. When SrTiO_3_ is irradiated with light of energy greater than its bandgap energy, electrons are excited to the conduction band from the valence band, thus creating electron–hole pairs (Equation 2). Generally, most of the photogenerated electrons and holes recombine rapidly, and only a few of them participate in redox reactions. It is noted that graphene, which is an excellent electron acceptor and conductor, has a Fermi level (-0.08 V vs. NHE [37]) positive to the conduction band potential of SrTiO_3_ (-0.84 V). When SrTiO_3_ particles are assembled onto graphene sheets, the photogenerated electrons can readily transfer from the conduction band of SrTiO_3_ to graphene (Equation 3). Thus, the recombination of electron–hole pairs can be effectively suppressed in the composites, which leads to an increased availability of electrons and holes for the photocatalytic reactions. The Fermi level of graphene is positive to the redox potential of O_2_/·O_2_ (-0.13 V vs. NHE) but negative to that of O_2_/H_2_O_2_ (+0.695 vs. NHE) [31,38]. This implies that the photogenerated e^-^ which transferred onto the graphene cannot thermodynamically react with O_2_ to produce · O_2_, but can react with O_2_ and H^+^ to produce H_2_O_2_ (Equation 4). H_2_O_2_ is an active species that can cause dye degradation, and moreover, H_2_O_2_ can also participate in the reactions as described in Equations 5 and 6 to form another active species · OH. The valence band potential of SrTiO_3_ (+2.51 V) is positive to the redox potential of OH^-^/·OH (+1.89 V vs. NHE) [39], indicating that the photogenerated h^+^ can react with OH^-^ to produce · OH (Equation 7). As a consequence, the active species · OH, h^+^, and H_2_O_2_ work together to degrade AO7 (Equation 8).

(2) $$\mathrm{SrTi}O_{3}+\mathrm{h\nu}\to \mathrm{SrTi}O_{3}\left(e^{-}+\;h^{+}\right)$$

(3) $$e^{-}+\mathrm{Graphene}\to \mathrm{Graphene}\;\left(e^{-}\right)$$

(4) $$\begin{array}{c} 2\;G\mathrm{raphene}\;\left(e^{-}\right)+\;O_{2}+\;2H^{+}\to 2\;\mathrm{Graphene}\\ \;+\;H_{2}O_{2} \end{array}$$

(5) $$H_{2}O_{2}+\mathrm{h\nu}\to 2\cdot \mathrm{OH}$$

(6) $$H_{2}O_{2}+\;e^{-}\to \cdot \mathrm{OH}+\;OH^{-}$$

(7) $$h^{+}+\;OH^{-}\to \cdot \mathrm{OH}$$

(8) $$h^{+},\;\cdot \mathrm{OH},\;\mathrm{or}\;H_{2}O_{2}+\mathrm{AO}7\to \mathrm{Degradation}\;\mathrm{products}$$

From Figure 6, it is found that the photocatalytic activity of the composites is highly related to the content of graphene, which can be explained as follows. With raising the graphene content, the amount of SrTiO_3_ particles decorated on the surface of graphene is expected to increase, thus providing more photogenerated carriers for the photocatalytic reaction. When the graphene content in the composites reaches 7.5%, the SrTiO_3_ particles are decorated sufficiently, consequently leading to the achievement of the highest photocatalytic activity. However, with further increasing graphene content above 7.5%, the photocatalytic efficiency begins to exhibit a decreasing trend. The possible reason is that the excessive graphene may shield the light and decrease the photon absorption by the SrTiO_3_ particles, and moreover, the amount of available surface active sites tends to be reduced due to an increasing coverage of graphene onto the surface of the SrTiO_3_ particles.

**Figure 10:**
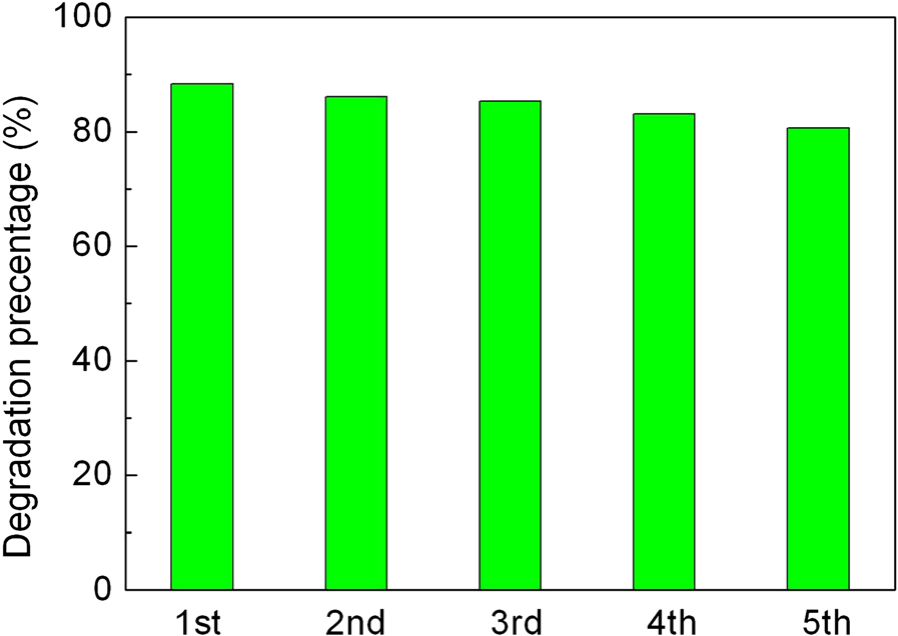
**Degradation percentage of AO7 after irradiation for 6 h over SrTiO**_**3**_**-graphene(7.5%) composites during the five photocatalytic cycles.**

**Figure 11:**
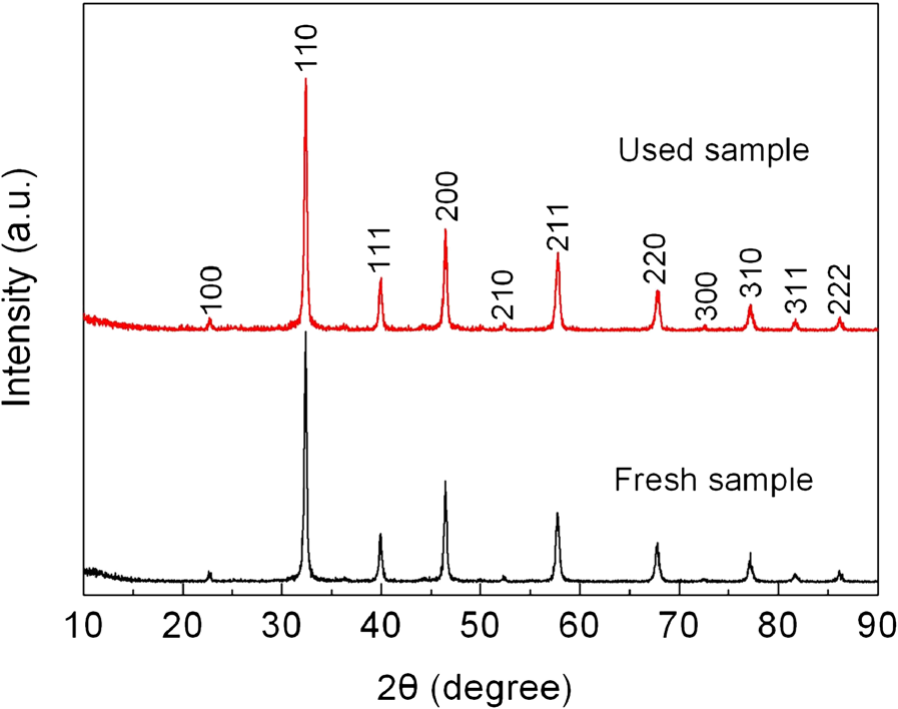
**XRD patterns of SrTiO**_**3**_**-graphene(7.5%) composites before and after the photocatalytic experiment.**

**Figure 12:**
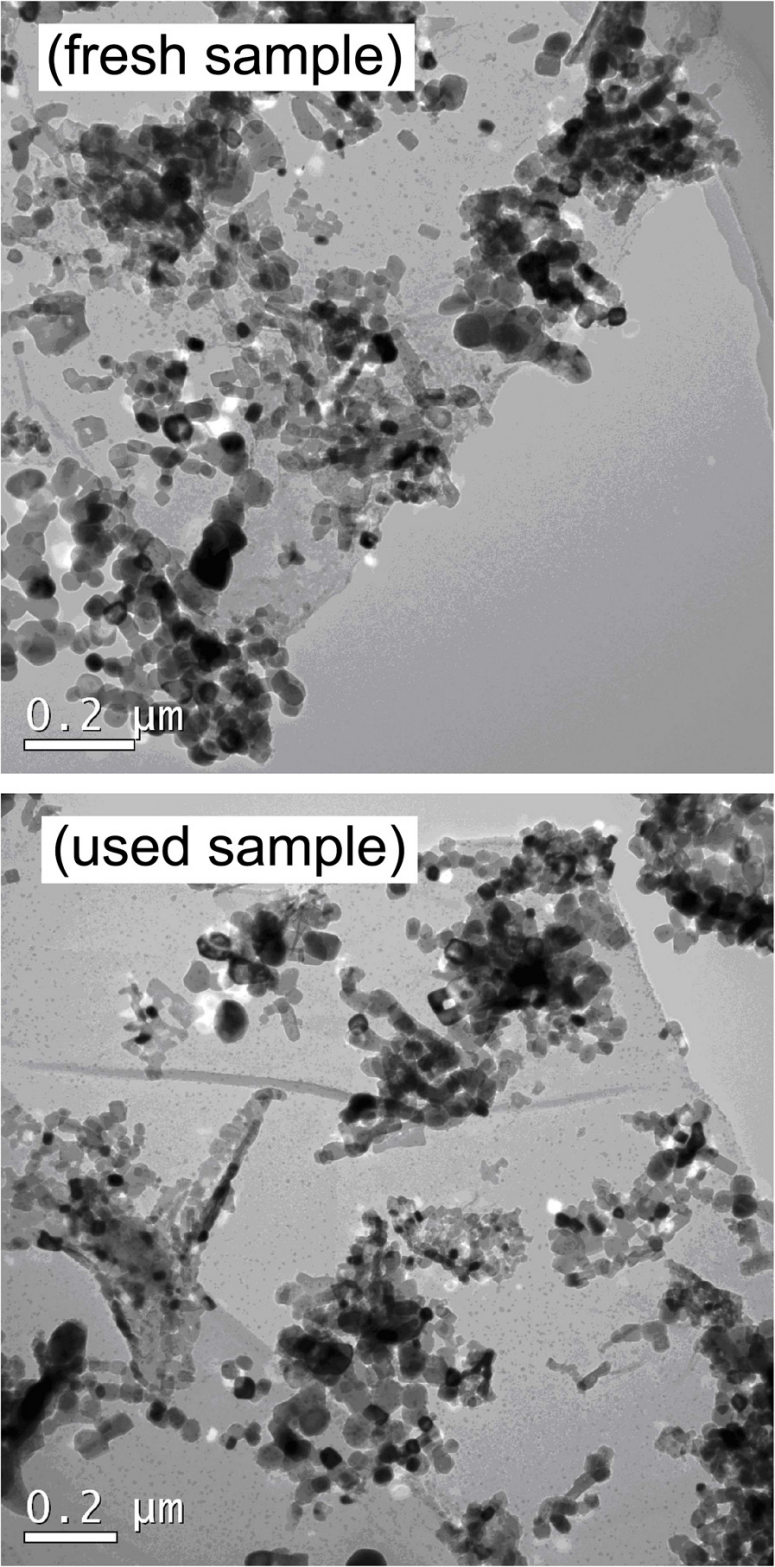
**TEM images of the SrTiO**_**3**_**-graphene(7.5%) composites before (top) and after (bottom) the photocatalytic experiment.**

Besides the photocatalytic activity, the reusability of photocatalysts is another crucial factor for their practical applications. The stability of the SrTiO_3_-graphene(7.5%) composites is examined by the recycling photocatalytic experiment, as shown in Figure 10. It reveals that the degradation percentage of AO7 maintains 80% to 88% for five consecutive recycles. The tiny or negligible lose of the photocatalytic efficiency indicates the excellent photocatalytic reusability of the as-prepared SrTiO_3_-graphene composites. Figure 11 shows the XRD patterns of the composites before and after the recycle experiment, revealing no obvious crystal structure changes. Figure 12 shows the TEM images of the composites before and after the recycle experiment, from which one can see that SrTiO_3_ particles are still well decorated on the graphene sheets.

## Conclusions

SrTiO_3_-graphene nanocomposites were prepared by irradiating the mixture solution of SrTiO_3_ nanoparticles and graphene oxide sheets, during which graphene oxide receives electrons from the excited SrTiO_3_ nanoparticles to be reduced to graphene, simultaneously leading to the assembly of SrTiO_3_ nanoparticles onto graphene sheets. Compared to the bare SrTiO_3_ nanoparticles, the as-prepared SrTiO_3_-graphene composites exhibit an enhanced photocatalytic activity for the degradation of AO7 under irradiation of UV light. This can be attributed to the effective separation of photogenerated electron–hole pairs due to the electron transfer from SrTiO_3_ to graphene and, hence, increased availability of electrons and holes for the photocatalytic reaction. The enhanced generation of · OH radicals is observed over the irradiated SrTiO_3_-graphene composites compared to the bare SrTiO_3_ nanoparticles. The photocatalytic efficiency is slightly deceased by purging with N_2_ but is significantly suppressed by the addition of ethanol and KI (especially for the latter). Based on the experimental results, ·OH, h^+^, and H_2_O_2_ are suggested to be the main active species causing the dye degradation.

## Abbreviations

AO: ammonium oxalate; AO7: acid orange 7; CB: conduction band; e^-^: photogeneration of electron; *E*_g_: bandgap energy; FTIR: Fourier transform infrared spectroscopy; h^+^: photogeneration of hole; H_2_O_2_: hydrogen peroxide; NHE: normal hydrogen electrode; OH: hydroxyl radicals; PL: photoluminescence; TA: terephthalic acid; TAOH: 2-hydroxyterephthalic acid; TEM: transmission electron microscope; UV: ultraviolet; VB: valence band; XRD: X-ray powder diffraction.

## Competing interests

The authors declare that they have no competing interests.

## Authors’ contributions

HY and TX conceived the idea of experiments. TX, LD, JM, and HZ carried out the preparation and characterization of the samples. HY, TX, and JD analyzed and discussed the results of the experiments. TX drafted the manuscript. HY improved the manuscript. All authors read and approved the final manuscript.

## Authors’ information

HY is a professor and a Ph.D. degree holder specializing in the investigation of photocatalytic and nanometer materials. JD is a professor and a Ph.D. degree holder specializing in the investigation of nanometer materials. JM and HZ are instructors and M.Sc. degree holders specializing in the research of nanometer materials. TX is a doctoral candidate major in the study of photocatalytic materials. LD is a graduate student major in the preparation of photocatalytic materials.
